# The Influence of Vesicle Shape and Medium Conductivity on Possible Electrofusion under a Pulsed Electric Field

**DOI:** 10.1371/journal.pone.0158739

**Published:** 2016-07-08

**Authors:** Linying Liu, Zheng Mao, Jianhua Zhang, Na Liu, Qing Huo Liu

**Affiliations:** 1 Institute of Electromagnetics and Acoustics, and Department of Electronic Science, Xiamen University, Xiamen, 361005, P.R. China; 2 Department of Electrical and Computer Engineering, Duke University, Durham, NC, 27708, United States of America; University of Zurich, SWITZERLAND

## Abstract

The effects of electric field on lipid membrane and cells have been extensively studied in the last decades. The phenomena of electroporation and electrofusion are of particular interest due to their wide use in cell biology and biotechnology. However, numerical studies on the electrofusion of cells (or vesicles) with different deformed shapes are still rare. Vesicle, being of cell size, can be treated as a simple model of cell to investigate the behaviors of cell in electric field. Based on the finite element method, we investigate the effect of vesicle shape on electrofusion of contact vesicles in various medium conditions. The transmembrane voltage (TMV) and pore density induced by a pulsed field are examined to analyze the possibility of vesicle fusion. In two different medium conditions, the prolate shape is observed to have selective electroporation at the contact area of vesicles when the exterior conductivity is smaller than the interior one; selective electroporation is more inclined to be found at the poles of the oblate vesicles when the exterior conductivity is larger than the interior one. Furthermore, we find that when the exterior conductivity is lower than the internal conductivity, the pulse can induce a selective electroporation at the contact area between two vesicles regardless of the vesicle shape. Both of these two findings have important practical applications in guiding electrofusion experiments.

## Introduction

The exposure of cells to a pulsed electric field can lead to a variety of responses. In all these responses, the phenomena of electroporation and electrofusion are of particular interest, because of their widespread application in cell biology and biotechnology [1, 2]. As charges of opposite polarities accumulate on both sides of the cell membrane, they produce a transmembrane voltage (TMV) and induce cell deformation. Once the TMV reaches a critical value, membrane poration occurs, leading to a substantial increase in the membrane permeability and introducing various molecules into cells. In the highly permeable state, cells in close contact can fuse. Due to its fusion efficiency, this electrofusion method is increasingly used in hybridoma technology [3, 4] and cancer vaccines [5, 6].

Vesicle, which consists of protein-free lipid bilayers, has been instrumental in modeling the conditions of bilayer fusion and defining the sequences of the intermediate structures formed in the course of bilayer merger [7–9]. In the last few years, experiments of giant vesicle fusion under direct current (DC) pulsed fields had been carried out by Dimova’s group [10, 11]. Interestingly, under the electric field, vesicles can deform from spherical shape to ellipsoidal shape [12–14]. In AC fields, spherical vesicles assume ellipsoidal shapes [13]. Depending on the applied field strength and frequency, and the conductivity values of the media, either prolate or oblate shape transition was observed [11, 12, 14]. In a DC pulsed field, vesicle deformations are expected to have similar behaviors under the same condition [12]. By controlling the appropriate amplitude, duration, and number of applied pulses, electroporation is obtained in a pulsed field [15, 16]. When one or both of the two physically contacting vesicles electroporates, electrofusion can be observed [17]. Obviously, understanding electroresponses under these shapes is critical to revealing the fusion mechanism and designing the high-efficiency fusion electric pulses.

However, detailed observation of the responses caused by an electric pulse on cells (or vesicles) is difficult because of the short duration of the pulse (microsecond or even nanosecond). Fortunately, numerical modeling, as a supplementary means of experiments, can simulate the electroresponses of cell (or vesicle) under a pulsed field, which is useful to reveal more details before fusion and to design optimized experiments. Neumann et al. have reported cell-cell electrofusion via a numerical model considering protein-protein interactions at the contact area of the fusing cells [9]. In recent years, the simplified cell models with single shell, double shells or multi-shells, have been used in the numerical and theoretical studies by ignoring the protein effects [18–28]. Using the steady-state electric field theory, analytical description of TMV induced by an electric field on spheroidal cells is possible under the assumption of a nonconductive membrane [18]. Base on this model, Techaumnat has analyzed the influence of cell geometry and the conductivity of the extracellular medium on the TMV of non-spherical biological cells under DC field using a boundary element method [19]. He predicted that the maximum TMV is found at the contact poles if the conductivity of the extracellular medium is very low. Another analytical model of the TMV for a spheroidal shell in AC electric field is based on simplified equations [20, 21]. By introducing an “influential radius” to gain the equivalent internal (external) electric field and converting these finite elements to a RC-lump model, they successfully obtain the TMV of arbitrarily oriented ellipsoidal [20] and cylindrical cells and reveal that the larger the shape elongation, the larger the maximum TMV [21, 22]. However, the electric field theory they used does not consider the influence of temporal variation [18, 19] or the influence of the dynamic process of pore generation on membrane [20–22].

Moreover, studies considering dynamic process of membrane pore generation have been performed. Hu and Joshi have reported self-consistent evaluation of both the TMV across the membrane of single spheroidal cell responses to ultrashort, high-intensity pulses coupling the Laplace equation with Smoluchowski theory of pore formation [23, 24]. They revealed that the oblate spheroids lead to a higher TMV and allow a great fraction of the surface area to be porated [23, 24]. Weaver and coworkers employed two spatially distributed two-dimensional cell models which are constructed using the meshed transport network method to investigate TMV and pore density for different organelles’ membrane of a cell. Their results show that sub-microsecond, megavolt-per-meter pulses can induce intracellular membrane electroporation while plasma membrane still keeps intact [25]. Pucihar and co-workers proposed a finite element model to study the electrofusion of a pair spherical cells with different sizes using nanosecond electric pulse [26–28]. They demonstrate that nanosecond pulses can induce selective electroporation of the contact areas between cells regardless of cell sizes [26–28].

However, the investigation of the influence of vesicle shape and medium conductivity on possible electrofusion under pulsed field in considering the dynamics of membrane pore generation has not been reported. In this work, we employ a finite element model to examine the electroresponses of a single vesicle and a pair of vesicles with different shapes (oblate, spherical and prolate) in a pulsed electric field to investigate the possibility of fusion. Since the solution conductivities of the internal and external of vesicles (*σ*_*i*_ and *σ*_*e*_) have a strong influence on the electroresponses [12], they are also varied in our simulations. Considering the time scale and the strength of the pulsed field used in the most common experiment, we choose a 1.5 kV/cm microsecond duration square pulse field. We investigate the influence of vesicle shapes on the TMVs and pore densities for single vesicle or a pair of vesicles in two medium conditions (*σ*_*i*_ < *σ*_*e*_, and *σ*_*i*_ > *σ*_*e*_). Meanwhile, the time evolution of pore density at the poles and contact area points (only for a pair vesicles) is analyzed. Our simulation demonstrates that when the conductivity of the internal vesicle solution is higher than the external one, selective electroporation for prolate vesicle at the contact area is observed. Interestingly, when the exterior medium conductivity is lower (e.g. 0.01 S/m, 0.001 S/m) than the inner one (0.2 S/m), the pore density at the contact area is larger than the pole points regardless the vesicle shape.

## Methods

**Fig 1:**
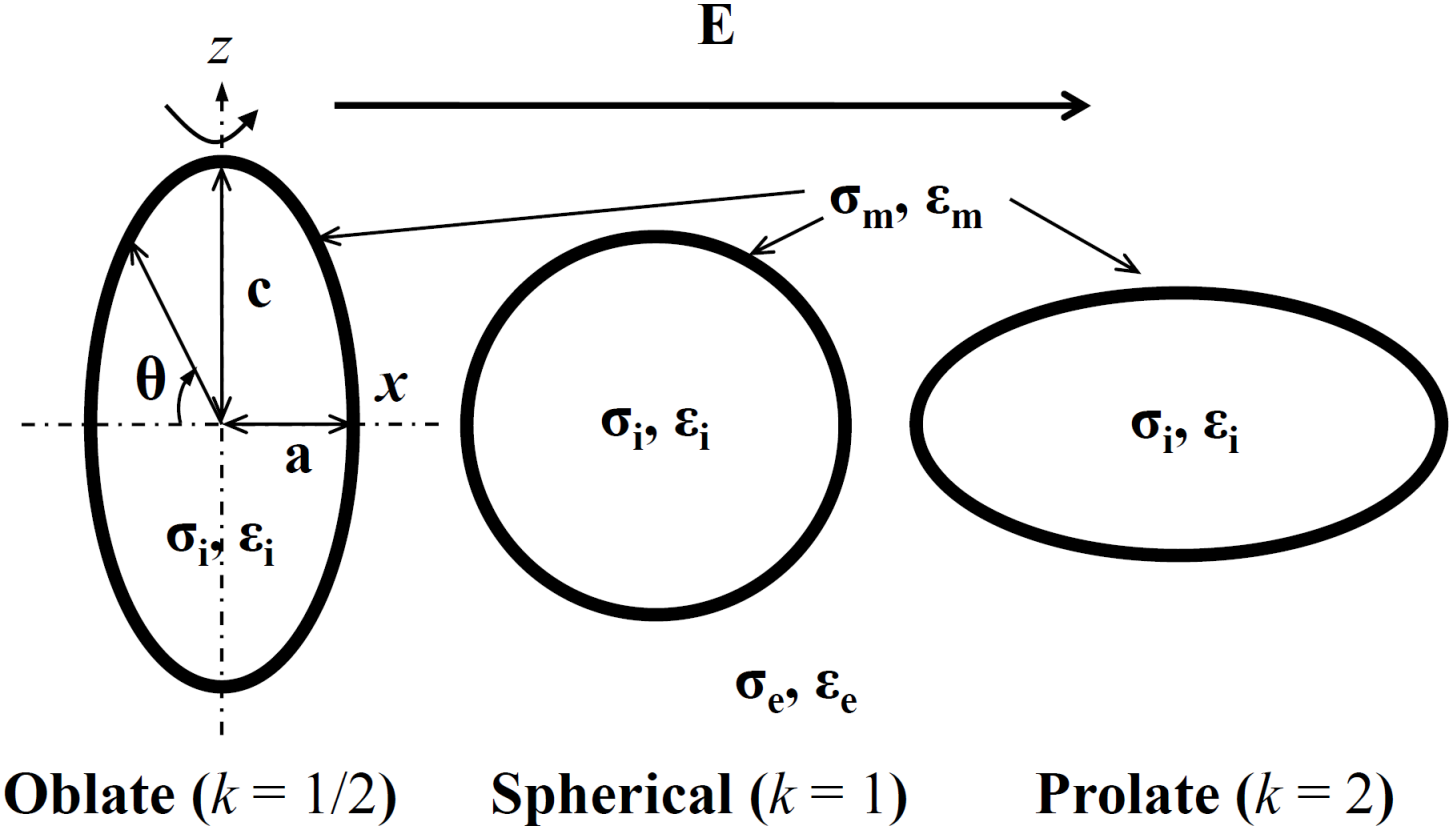
Variations of vesicle geometries used for analysis. The dash lines in the oblate vesicle represent its axes *x* and *z*. The applied external electric field is along the *x*-axis of the vesicles. The axis ratio (*k* = *a*/*c*) used here is equal to 1/2, 1, and 2, respectively, and *θ* is the polar angle, measured along the clockwise direction. *σ*_*i*_, *σ*_*m*_, *σ*_*e*_ and *ε*_*i*_, *ε*_*m*_, *ε*_*e*_ are the conductivities and permittivities of internal solution, membrane and external solution.

In general, a spheroidal vesicle has three axes, namely *a*, *b*, *c*. In the following we will restrict our consideration to the simplified case of spheroidal vesicles (*a* = *b*) and consider only two-dimensional axisymmetric model (including axis *a*, *c*). As shown in Fig 1, the external electric field *E* is taken to be perpendicular to the *z*(*c*) axis, the axis of revolution which, when rotated, corresponds to a three-dimensional model. The volume of a spheroidal vesicle ($V=\frac{4}{3}\pi a^{2}c$) is set to be constant whenever its shape becomes prolate or oblate. Here we set the axis ratio *k* = *a*/*c* to describe the vesicle shapes. With a fixed volume 4200 *μm*^3^, (equal to the volume of spherical vesicle with *a* = *c* = 10 *μm*), the vesicles investigated in our calculations have three different axis ratios *k* = 1/2, 1, 2, which represent oblate spheroidal, spherical, and prolate spheroidal shapes, responsively. Fig 1 shows the schematic of the vesicles with different shapes suspending in a medium.

**Fig 2:**
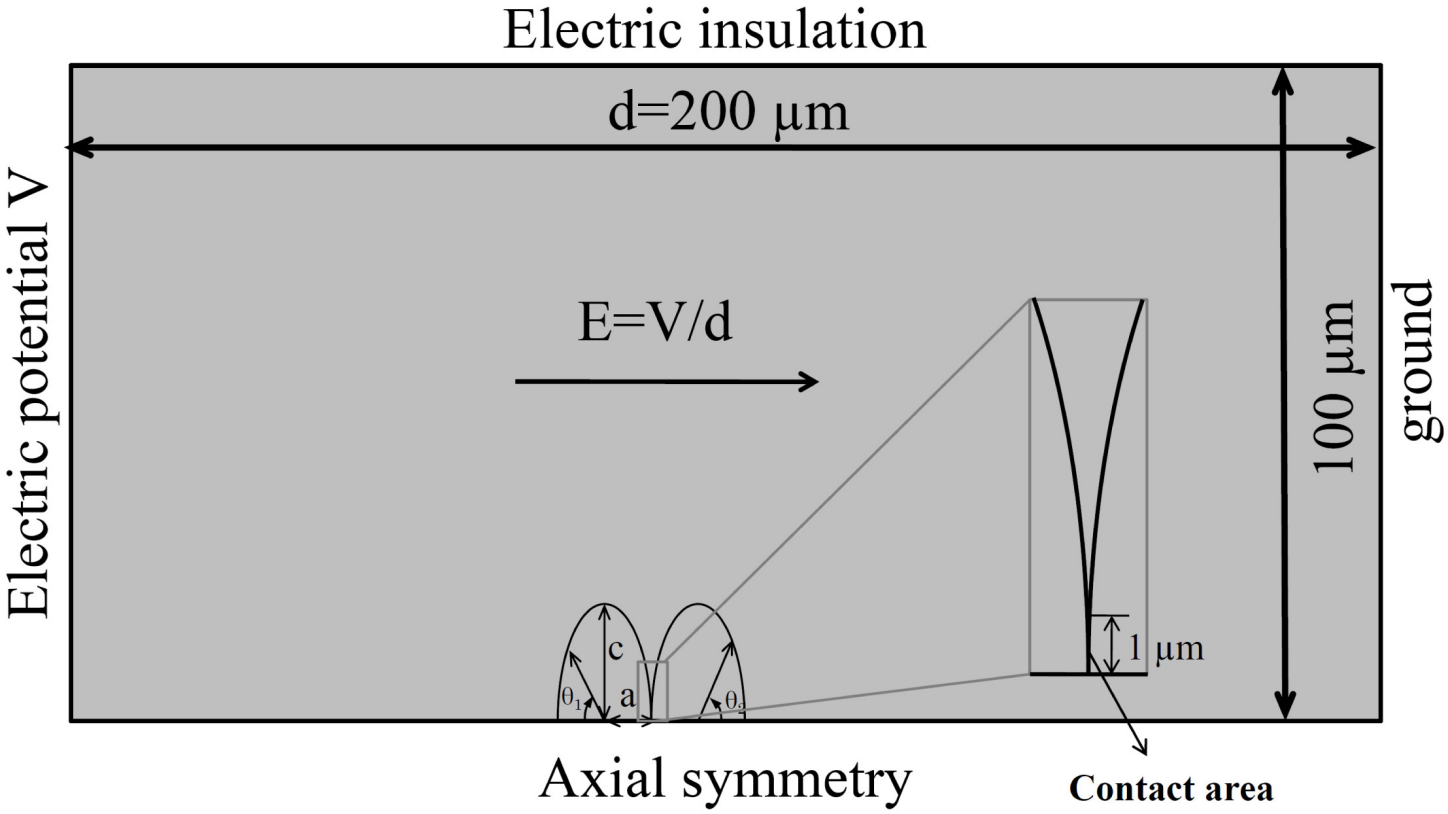
Model of two contact vesicles with axis ratios (*k* = 1/2) exposed to an electric field. The axis ‘a’ is parallel to the direction of electric field (also the axis of symmetry). The magnitude of electric field (1.5 kV/cm) is determined as the potential difference between the two electrodes (electrode potential), divided by the electrode distance. The direction of the electric field is indicated with an arrow. Polar angle *θ*_1_ for the left vesicle arises along the clockwise direction, whereas polar angle *θ*_2_ for the right vesicle arises along the counterclockwise direction.

The two-dimensional axisymmetric model of two vesicles (with the axis ratio defined above) is constructed in Comsol Multiphysics 4.4 (Comsol) using the finite element method. The two vesicles in contact aligned in the direction of the external electric filed are placed in a cylinder of radius 100 *μm* and height 200 *μm* representing the extracellular medium (see Fig 2 for the cross section). The left side of the cylinder is modeled as an electrode with its electric potential given by a pulse function, which is realized by a ramp functions (Comsol function *rm*1) with 1 *μs* rise time, and our simulation time starts from the end of the rise time. The right side of the cylinder is modeled as ground. The outer radial surface is set as electric insulation and the bottom has the axial symmetry condition. The bounding box is much larger than the vesicle so that the boundary effects would be negligible. The vesicles are positioned one next to another with part of their membrane forming a contact area. The length of the contact area is set to 1 *μm* (Fig 2 zoom-in box) and the thickness of the contact area is twice the other part of the membrane.

The numerical model uses a system of equations provided by Comsol Multiphysics (*AC/DC module, Electric Currents, Time Dependent Study*). The electric potential *V* in each subdomain of the model is determined by equation

$$\begin{array}{c} -\nabla \cdot \left(\sigma_{k}\nabla V\right)-\nabla \cdot \frac{\partial \varepsilon_{k}\nabla V}{\partial t}=0 \end{array}$$ (1)

where *σ*_*k*_ and *ε*_*k*_ denote the conductivity and dielectric permittivity of a given subdomain (internal medium, membrane and external medium, respectively). Instead of physically included the membrane in the model, we replace it by a boundary condition known as the *Contact Impedance* [29, 30]:

$$\begin{array}{c} n\cdot J=\frac{\sigma_{m}}{d_{m}}\left(△V\right)+\frac{\varepsilon_{m}}{d_{m}}\frac{\partial △V}{\partial t} \end{array}$$ (2)

Here, **n** is the outward unit vector normal to the boundary surface, **J** is the electric current density, and △*V*, *σ*_*m*_, *ε*_*m*_ and *d*_*m*_ are the potential difference, conductivity, permittivity, and thickness of the membrane, respectively. It should be noted that *ε*_*m*_ and *d*_*m*_ are constant parameters as set in Table 1. Moreover, *σ*_*m*_ is related to the dynamics of pore generation. Here, we do not take into account the ionic current [31], since it has negligible influence on the results [26].

**Table 1:** Parameters used in calculation.

Symbol	Quantity	Value*
*r*_*p*_	pore radius	0.76 nm
*d*_*m*_	membrane thickness	5 nm
*σ*_*p*_	pore conductivity [30]	(*σ*_*e*_ − *σ*_*i*_)/ln(*σ*_*e*_/*σ*_*i*_) S/m
*σ*_*i*_	interior conductivity	0.2 S/m
*σ*_*m*_	membrane conductivity	5 × 10^−7^ + *σ*_*ep*_ S/m
*σ*_*e*_	exterior conductivity	10^−3^ ∼ 1 S/m
*ε*_0_	vacuum permittivity	8.85 × 10^−12^ F/cm
*ε*_*i*_	interior permittivity	80 *ε*_0_
*ε*_*m*_	membrane permittivity	4.5 *ε*_0_
*ε*_*e*_	exterior permittivity	80 *ε*_0_
*q*	electroporation constant	2.46
*α*	electroporation parameter	10^9^ m^−2^s^−1^
*V*_*ep*_	characteristic voltage	0.258 V
*N*_0_	equilibrium pore density	1.5 × 10^9^ m^−2^

*Values are mostly from G. Pucihar et al. [26], except the conductivity of internal and exeternal solution.

The dynamics process of pore generation is also considered in our model, which is governed by the following differential equation [31],

$$\begin{array}{c} \frac{dN(t)}{dt}=\alpha e^{{\left(△V\left(t\right)/V_{ep}\right)}^{2}}\left(1-\frac{N(t)}{N_{0}}e^{-q{\left(△V\left(t\right)/V_{ep}\right)}^{2}}\right) \end{array}$$ (3)

where *N* denotes the induced pore density in the membrane, *N*_0_ is the equilibrium pore density, and *α*, *q* and *V*_*ep*_ are constants (listed in Table 1). Eq (3) is coupled into Comsol with the *Weak Form Boundary PDE* application mode [30].

The inducement of pores alters the membrane conductivity. The dynamically changed membrane conductivity *σ*_*ep*_ due to the electroporation can be described by the equation [31, 32]:

$$\begin{array}{c} \sigma_{ep}=N\left(t\right)\frac{2\pi r_{p}^{2}\sigma_{p}d_{m}}{\pi r_{p}+2d_{m}} \end{array}$$ (4)

where *r*_*p*_ and *σ*_*p*_ are the radius and conductivity of a single pore [32], and *σ*_*ep*_ is proportional to pore density N. The total membrane conductivity *σ*_*m*_ was calculated at each time step as the sum of the passive membrane conductivity and the conductivity due to electroporation *σ*_*ep*_. Parameters used for theoretical calculation are shown in Table 1.

## Results and Discussions

Basically, the external field produces a TMV, which can lead to pore formation in the membrane. This pore density modulates the local conductivity of membrane, thereby affecting the local fields and subsequently the development of TMV. Since the electroporation correlates with the occurring of vesicle fusion if the two pored vesicles are in close physical contact, we calculate the electroporation behaviors of the contact vesicles to predict their possible fusion.

In the following sections, we will describe the responses of different spheroidal vesicles when subjected to a microsecond square pulsed field. We first consider the TMV and the possible electroporation of the single vesicle, with oblate, sphercial and prolate shapes, in the medium condition *σ*_*i*_ < *σ*_*e*_ (*σ*_*i*_ = 0.2 S/m, *σ*_*e*_ = 1 S/m) and *σ*_*i*_ > *σ*_*e*_ (*σ*_*i*_ = 0.2 S/m, *σ*_*e*_ = 0.01 S/m); the other parameters are listed in Table 1. Then under the same condition we couple two vesicles with the same shapes to investigate the possible vesicle fusion. Finally, the influence of the exterior conductivity, ranging from 10^−3^ S/m to 1S/m, on the possible electrofusion of the oblate and prolate vesicles as a function of time is discussed. Moreover, to ensure that the results generated from this numerical model are reliable, we calculate TMV and pore density for a single or pair vesicles with the same parameters as previous work (Refs. [19, 25, 26]), and compare our results with them. We find that our simulation results are in good agreement with all the previous works mentioned (Refs. [19, 25, 26]).

### The influence of vesicle shapes on electroporation and possible electrofusion

#### Single vesicle response to a pulsed field

Before investigating the electrofusion behaviors of the contact paired vesicles, we study the electroporation of a single vesicle under the condition that *σ*_*i*_ < *σ*_*e*_ (*σ*_*i*_ = 0.2 S/m, *σ*_*e*_ = 1 S/m) and *σ*_*i*_ > *σ*_*e*_ (*σ*_*i*_ = 0.2 S/m, *σ*_*e*_ = 0.01 S/m) to validate whether the computation method we used is reliable. As we all know, cells exposed to pulse electric field can be charged and the charge time is given by

$$\begin{array}{c} t_{c}=RC_{m}\left(\frac{1}{\sigma_{i}}+\frac{1}{2\sigma_{e}}\right) \end{array}$$ (5)

where *R* is the vesicle radius and *C*_*m*_ is the membrane capacitance ($C_{m}=\frac{\varepsilon_{m}\varepsilon_{0}}{d_{m}}∼$ 0.008 F/m^2^) [33]. Thus we can estimate that the charge time is 0.44 *μs* *σ*_*i*_ < *σ*_*e*_ (*σ*_*i*_ = 0.2 S/m, *σ*_*e*_ = 1 S/m) and 4.4 *μs* *σ*_*i*_ > *σ*_*e*_ (*σ*_*i*_ = 0.2 S/m, *σ*_*e*_ = 0.01 S/m).

**Fig 3:**
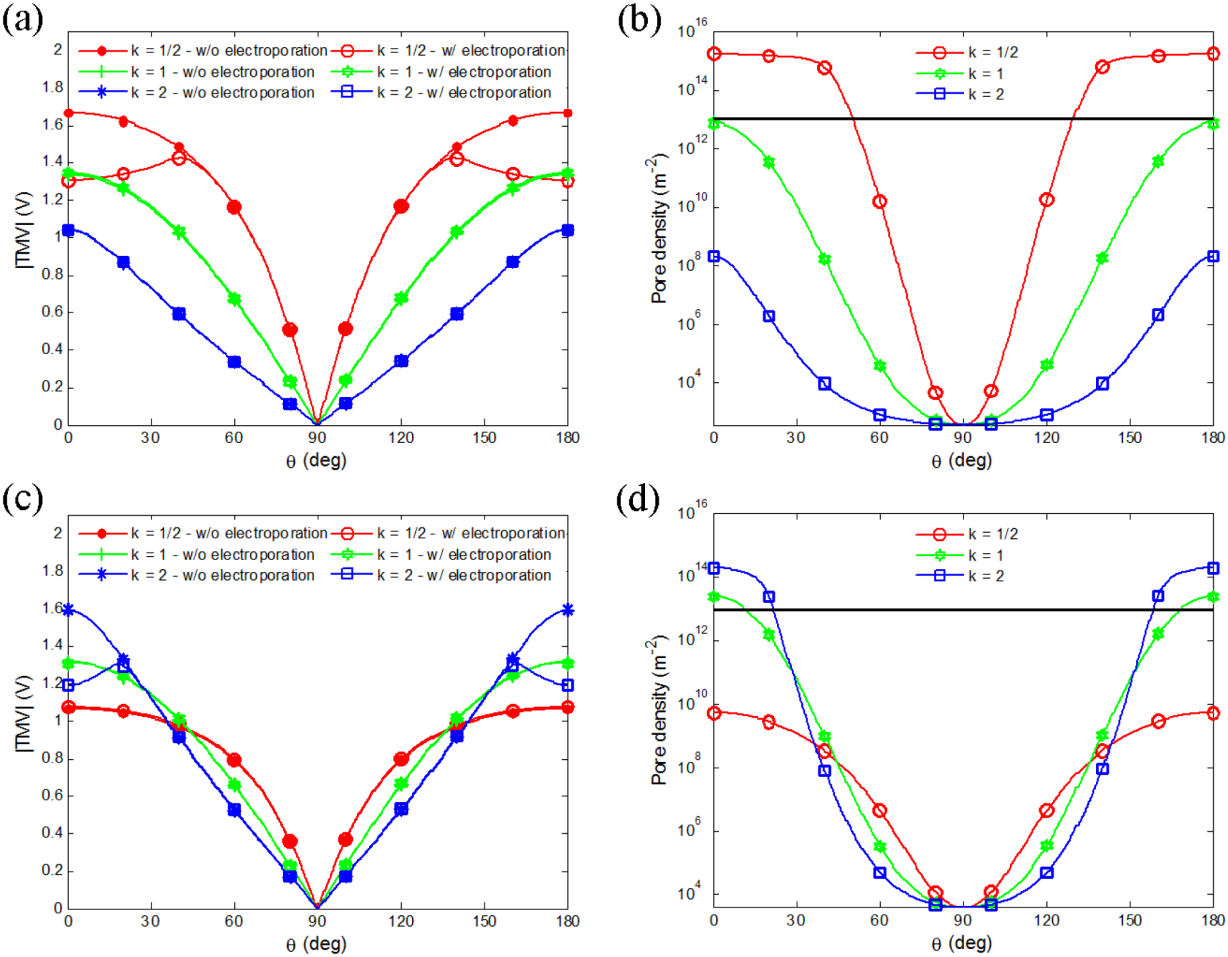
(Color online) **TMV and pore density versus central angle (*θ*, from left to right) for oblate (*k* = 1/2), spherical (*k* = 1), and prolate (*k* = 2) vesicles in medium with conductivity 1 S/m at *t* = 0.4 *μs* (a, b) and 0.01 S/m at *t* = 4 *μs* (c, d)** with electric field of 1.5 kV/cm. The dash color lines (in a, c) represent TMV without eletroporation and the corresponding solid color lines represent TMV with electroporation. Thin horizontal lines in (*b*) and (*d*) indicate a pore density of 10^13^ *m*^−2^.

As charges accumulate at the bilayer interface, they produce a transmembrane voltage (TMV). When the TMV reaches sufficiently high value (∼ 1 V), a large number of conductive pores form in the membrane (membrane is electroporated) and membrane conductivity can increase by several orders of magnitude (significant electroporation occurs). Coupling the dynamic process of pore generation on membrane, the electroporation-related time-dependent conductivity changes of the membrane will eventually affect the TMV, as shown in Fig 3. This simulation result shown is obtained by considering a single spheroidal vesicle with the axis ratios mentioned above exposed to a microsecond pulsed field in the two medium conditions. The TMV of vesicle without or with electroporation situation is examined to reveal the influence of time-dependent pore generation on TMV. In the absence of electroporation model, the membrane conductivity is constant, whereas in the electroporation model, the membrane conductivity is dynamically changed. Obviously, the changeable membrane conductivity will affect the TMV.

Fig 3 shows the TMV and the pore density of the single vesicle in the two medium conductivity values at *t* = 0.4 *μs* (Fig 3a and 3b) and *t* = 4 *μs* (Fig 3c and 3d). The time is chosen to ensure that the significant electroporation of membrane can be observed at least in one shape of vesicles, which approaches the end of charge time (0.4 *μs*, and 4 *μs* respectively). Generally, the pore density of a value 10^13^ m^−2^ (10 pores per *μm*^2^) is used to represent the significant electroporation in many literatures [26, 31, 34].

The dash color lines in the Fig 3a and 3c represent TMVs without electroporation and the corresponding solid color lines represent TMVs with electroporation. As shown in the Fig 3a, for *σ*_*i*_ < *σ*_*e*_, the TMV of the oblate vesicle at poles is much higher than other shape vesicles (marked with filled circles). Whereas for *σ*_*i*_ > *σ*_*e*_ (Fig 3c), the TMV of prolate vesicle at poles exceeds other shape vesicles (marked with asterisks). In Fig 3, we can also observe that the TMV at the poles of oblate (prolate) shape vesicle when *σ*_*i*_ < *σ*_*e*_ (*σ*_*i*_ > *σ*_*e*_) with the electroporation (marked with empty circles and squares, respectively) has a much lower value than the one without the electroporation (marked with filled circles and asterisks, respectively). That is due to electroporation at the poles resulting in a higher conductivity (greater permeability) at the membrane which induces TMV dropping in the areas with high pore densities. As shown in Fig 3b and 3d, the pore density reaches its maximum at the poles of oblate (prolate) shape vesicle when *σ*_*i*_ < *σ*_*e*_ (*σ*_*i*_ > *σ*_*e*_) (marked with empty circles and squares, respectively), which exceeds the significant electroporation value. As a result, electroporation occurs at the poles of membrane and a reduction of TMV is found at the same area.

The simulation results are similar to the experimental observations made by Dimova et al., who use a fast imaging digital camera to record the deformation and poration behaviors of vesicles exposed to AC or DC pulsed field [11, 12, 35–37]. First, they report that in AC field or DC pulsed field, the spherical shape vesicles deform to oblate (prolate) when *σ*_*i*_ < *σ*_*e*_ (*σ*_*i*_ > *σ*_*e*_) [12, 36, 37]. Our work suggests that the electroporation of membrane can be easily observed at the poles of the oblate vesicle when *σ*_*i*_ < *σ*_*e*_ and the prolate vesicle when *σ*_*i*_ > *σ*_*e*_. Second, they observe that electroporation occurs at poles of prolate shape vesicles after applying a 200 *μs* DC pulse of strength E = 1.25 kV/cm to E = 2 kV/cm on egg-PC vesicles (or egg vesicle with different membrane component) when *σ*_*i*_ > *σ*_*e*_ [11, 12, 35], similar to the modeling results that show electroporation occurring at poles of prolate vesicle (Fig 3d marked with empty squares). In addition, Hu and Joshi have used a self-consistent numerical model to evaluate the TMV and pore generation on a single spherical cell, and also find electroporation occurred at the poles of oblate cell first when *σ*_*i*_ > *σ*_*e*_ [23, 24], which is in agreement with our results (shown in Fig 3b marked with empty circles).

#### Pair vesicles response to a pulsed field

**Fig 4:**
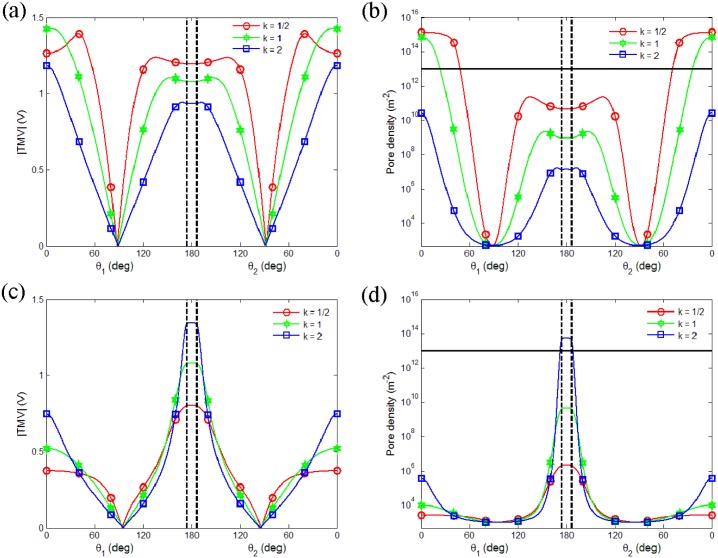
Calculation of TMV of two equally sized spheroidal vesicles with different axis ratios after the onset of exposure to a 1.5 kV/cm pulsed electric field. The results show that TMV and pore density as a function of central angle (left vesicle *θ*_1_, right vesicle *θ*_2_), in the medium condition *σ*_*i*_ < *σ*_*e*_ (*σ*_*i*_ = 0.2 S/m, *σ*_*e*_ = 1 S/m) at *t* = 0.5 *μs* (a, b) and *σ*_*i*_ > *σ*_*e*_ (*σ*_*i*_ = 0.2 S/m, *σ*_*e*_ = 0.01 S/m) at *t* = 1 *μs* (c, d). The contact areas are marked with the vertical line. Thin horizontal lines in (*b*) and (*d*) indicate a pore density of 10^13^ *m*^−2^.

Above results confirm that the method we used is reliable to study the response of vesicle under pulsed field. Fig 4 shows the TMV and pore density of pair vesicles with different axis ratios as a function of central angle of the left vesicle and the right vesicle (the angle around 180 deg represents contact area) when *σ*_*i*_ < *σ*_*e*_ (*σ*_*i*_ = 0.2 S/m, *σ*_*e*_ = 1 S/m, Fig 4a and 4b) and *σ*_*i*_ > *σ*_*e*_ (*σ*_*i*_ = 0.2 S/m, *σ*_*e*_ = 0.01 S/m, Fig 4c and 4d).

When *σ*_*i*_ < *σ*_*e*_, the TMV at the two poles along the electric field direction is bigger than that at the contact area between vesicles (Fig 4a). The corresponding pore density also gets its maximum at the poles and exceeds the significant electroporation value first (Fig 4b). Therefore, electroporation at the poles occurs first, whereas the membrane at the contact area still stays unruptured. As shown in Fig 4a and 4b, we can also find that the TMV of the oblate vesicle (marked with empty circles) is much higher than that of other shapes, except the porated area, where the TMV has a reduction due to the membrane permeability induced by the formed pores. Conversely, when *σ*_*i*_ > *σ*_*e*_, the TMV at the contact area between vesicles is bigger than that at the two poles along the electric field direction (Fig 4c). Correspondingly, electroporation of the membrane at the contact area occurs before the poration at the poles (Fig 4d). It indicates that the applied external pulsed field can induce the electroporation of two close vesicles at their contact area when the other parts remain intact. It makes the fusion of these two close vesicles to be possible.

As was well known, there are two necessary steps for fusion: (1) cells (or vesicles) are first in close physical contact; (2) cell (or vesicle) membranes are brought to fusogenic states [38]. So if the contact area of two close cells (or vesicles) is highly porated, in other words, the fusogenic states are formed, as the result, the fusion of the close cells (or vesicles) may be more easily. Hence, as mentioned above, the pore density shown in Fig 4d (marked with empty square) predicts that electrofusion of prolate pair vesicles may more easily happen when *σ*_*i*_ > *σ*_*e*_. Though the electrofusion is a post pulse phenomenon, our predictions are in agreement with experimental observation of pair vesicles exposed to a DC pulsed field. Using a fast imaging digital camera, fusion of two prolate deformable vesicles was observed when *σ*_*i*_ > *σ*_*e*_ [10, 11].

### The influence of medium conductivity on possible electrofusion

Inspired by the above results, we find that the solution conductivity has a strong influence on electroporation and possible electrofusion. Actually, an external solution with a conductivity varying from a few 10^−3^ S/m to 1 S/m is used in most vesicle fusion experiments [11, 12, 36]. In the present work, four different values: 1 S/m, 0. 1 S/m, 0.01 S/m and 10^−3^ S/m are chosen as the exterior solution conductivity in our calculations and the other parameters are set the same as in Table 1. Since the pore densities reach their maximum values at the contact area or at the poles along the electric field direction, calculation of time evolution of pore density at the pole and contact points for two oblate (*k* = 1/2) and prolate (*k* = 2) vesicles is performed. As the poles of two equally shaped vesicles are symmetric, we only plot out the pore density for the pole point in the left vesicle. If the point at contact area is first electroporated, we can predict that the vesicles in contact have a significant possibility to fuse.

**Fig 5:**
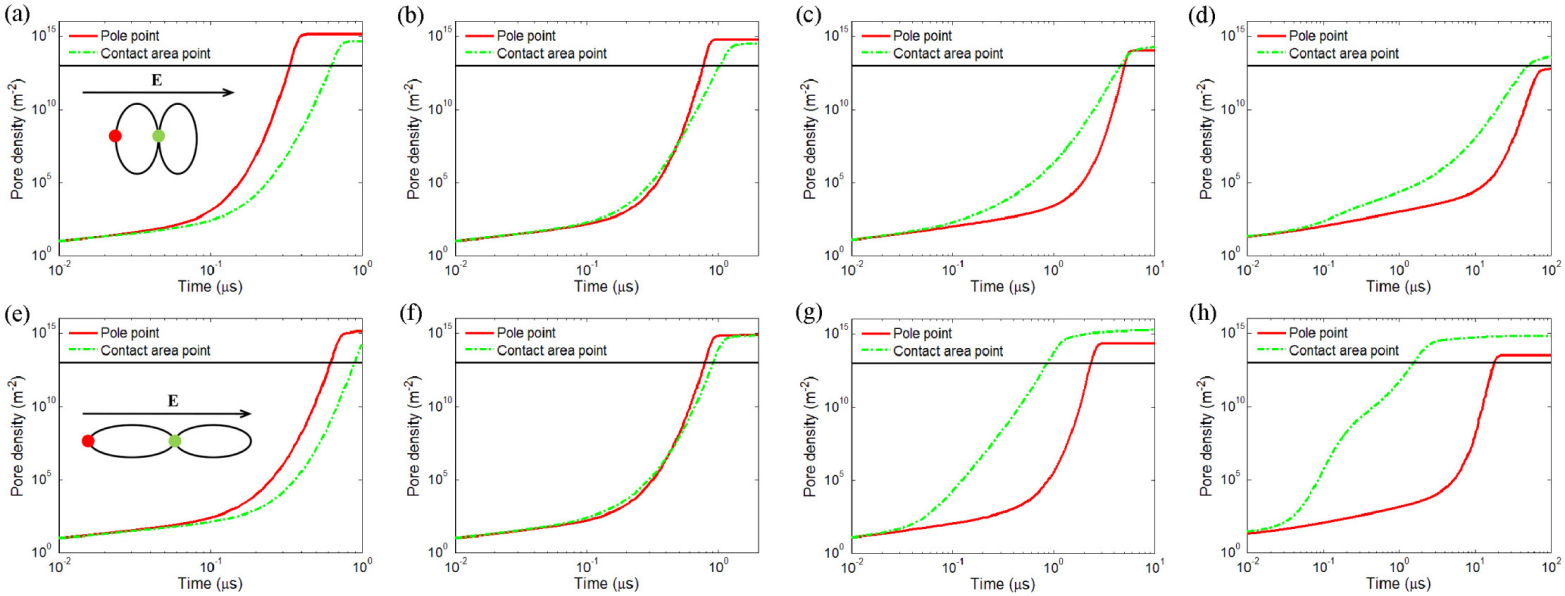
Calculated time evolution of pore density at the point of pole and at the contact area between two equally oblate shape vesicles (a to d) and prolate shape vesicles (e to g) in a pulsed electric field with 1.5 kV/cm. The pulse duration time limits to 1 *μs*, 2 *μs*, 10 *μs*, and 100 *μs* with corresponding external medium conductivity 1 S/m (a, e), 0.1 S/m (b, f), 0.01 S/m (c, g), and 10^−3^ S/m (d, h), respectively. Note that the time is presented on a logarithmic scale as this allows one to study the pore generation in the nanosecond and microsecond range simultaneously. The thin horizontal lines indicate a pore density of 10^13^ *m*^−2^.

Results in Fig 5 are the time course of pore density at the vesicle pole (solid red line) and the point in the middle of contact area (dash green line) of the oblate vesicles (Fig 5a–5d) and the prolate vesicles (Fig 5e–5g). Calculations are performed for a medium with four different conductivities (*σ*_*e*_): 1 S/m, 0.1 S/m, 0.01 S/m, and 10^−3^ S/m with pulse duration 1 *μs*, 2 *μs*, 10 *μs*, and 100 *μs*, respectively.

Obviously, the lower the external medium conductivity, the longer time it takes to obtain electroporation. When the conductivity of the external medium is larger than the internal medium (*σ*_*i*_ < *σ*_*e*_, *σ*_*e*_ = 1 S/m, Fig 5a and 5e), no matter what shape (oblate or prolate) the vesicle has, the pore density at the pole is bigger than that at the contact area between vesicles. This trend extends to the case when the conductivity of the external medium is similar to the internal medium (*σ*_*i*_ ≈ *σ*_*e*_, *σ*_*e*_ = 0.1 S/m, Fig 5b and 5f), however, to a lesser extent and for a short of period time (start from 0.4 *μs*). Therefore, electroporation occurs more easily at the pole than at the contact point when the conductivity of the external medium is higher than the internal medium. To some extent, the higher the conductivity of the external medium, the faster the electroporation at the pole than at the contact point. On the contrary, when the conductivity of the external medium is lower than the internal medium (*σ*_*i*_ > *σ*_*e*_, *σ*_*e*_ = 0.01 S/m, Fig 5c and 5g and *σ*_*e*_ = 10^−3^ S/m, Fig 5d and 5h), no matter what shape (oblate or prolate) the vesicle has, the pore density at contact area between vesicles is bigger and faster to exceed the significant electroporation value than that at the pole. And with the decreasing of the conductivity of the external medium, this trend seems more obvious.

The above results suggest that for a certain range of pulse durations, selective electroporation of contact areas between vesicles could be achieved. When the conductivity of the external medium is smaller than the internal medium, electroporation occurs first at the contact area, whereas the membrane at the pole is still unbroken. This presents the possibility for effective fusion of ellipsoidal shape vesicles without causing any damage to areas apart from the contact area. However, when the conductivity of the external medium is larger than the internal medium, electroporation occurs first at pole, conversely, the membrane at the contact area remain intact. It indicates that the external electric field may cause huge damages to vesicles before the possible fusion.

In fact, Pucihar and co-workers have demonstrated that using nanosecond pulsed field can obtain higher fusion probability regardless the cell size in the medium with *σ*_*e*_ = 0.01 S/m [26–28]. Compared with their work, our simulations have following novelties: first, the electroporation behaviors of ellipsoidal vesicles are firstly studied via the finite element method. Second, we first reveal the importance of the low exterior conductivity condition in electrofusion regardless the vesicle shape and predict the advantage of prolate shape vesicles in electrofusion. Meanwhile, the time evolution of pore density at the poles or at the contact points visually shows the dynamic process of pore generation on vesicle, which reveals more details before fusion. In experiments, spherical vesicles assume oblate (prolate) shape in the *σ*_*i*_ < *σ*_*e*_ (*σ*_*i*_ > *σ*_*e*_) condition as a response to the external field [12, 36, 37], but our model of both vesicle shapes in *σ*_*i*_ < *σ*_*e*_ or *σ*_*i*_ > *σ*_*e*_ still has universal significance. Some cells are naturally spheroidal. For example, mammalian red blood cells are close to an oblate spheroidal shape [39]; retina photoreceptor cells [40], many bacteria (such as *E*.*coli*, *Pseudomonas* [41]) and yeasts [42] roughly have a prolate spheroidal geometry.

In addition, our conclusion of the advantage of low exterior conductivity solution in electrofusion is in good agreement with Techaumnat’s work. He used the boundary element method to simulate the TMV of spheroidal cells under DC field [19] and predicted that the maximum TMV is found at the contact area if the conductivity of the extracellular medium is very low [19]. Moreover, the low exterior conductivity solution is quite preferable used in practical applications. Because the highly conductive medium produces Joule heating during exposure to external electric pulse if pulse durations in micro- to millisecond are used [43]. On the other hand, the extra-low conductivity is required for pearl-chain formation if dielectrophoresis is used for cell alignment [44].

However, in the present work, the shape of vesicle is assumed to be stationary during the whole process of exposure to an electric field. Under this assumption, or if the vesicle has small deformation within the pulse duration, our calculations can capture the essential characteristics of the vesicle response. If the vesicle deformation is large, however, our calculations may have some limitations. If large vesicle deformation is taken into account, it may introduce a higher value of pore density at both of their poles than the stationary spheroidal vesicles. The influence of the vesicle dynamic deformation on electroporation and electrofusion will be elucidated in the future research.

## Conclusion

In conclusion, in order to understand the response of vesicles to the external pulsed electric fields, the electroporation and electrofusion of vesicles with spheroidal shapes in various conductivity media are calculated by a finite element method. The calculations show that the electroporation of vesicle can be influenced by their shape and the medium conditions. The TMV and pore density reach their highest values at the poles of single oblate (prolate) vesicle when the medium conductivity ratio *σ*_*i*_ < *σ*_*e*_ (*σ*_*i*_ > *σ*_*e*_). It suggests that the electroporation easily occurs at the poles of oblate (prolate) vesicle when *σ*_*i*_ < *σ*_*e*_ (*σ*_*i*_ > *σ*_*e*_).

In the studies of the influence of vesicle shape on electrofusion, two equal vesicles with different shapes are brought to close contact in different medium conditions under a pulsed field. Their TMV and pore denstiy are calculated to examine the possibility of fusion. Our simulation results show that electroporation can easily occur at the poles of the oblate vesicles when *σ*_*i*_ < *σ*_*e*_ and at the contact areas of the prolate vesicles when *σ*_*i*_ > *σ*_*e*_. As we mentioned above, cells can fuse in the highly porated contact area. So the above results can reveal that electrofusion have greater probability occurring at the prolate pair vesicles when *σ*_*i*_ > *σ*_*e*_. Through the calculations of paired vesicles in various medium conditions, we can also demonstrate that the low exterior conductivity condition can induce selective electroporation at the contact areas between vesicles.

Using microsecond pulsed field we obtain a higher possibility of fusion regardless of the vesicle shape when conductivity of external medium is lower than that inside the vesicle. This needs to be stressed that it has practical applications in the control of electrofusion process in experiments by changing the conductivity of the medium. Moreover, the majority of results reported in this study are in good agreement with previous theoretical studies and experimental observations. The results reported in this work demonstrate that electromagnetic calculations with the finite element method provide a useful technique for studying the response of biological vesicles to an external field. Furthermore, our simulations about vesicle can also give some important information about cell fusion.
